# Notes and News

**Published:** 1893-01-21

**Authors:** 


### NOTES AND NEWS.

COLLECTED AND COMMUNICATED.

AT a conference of authorities of leading London hospitals, held by invitation of Lord Sandhurst at Spencer House on the 22nd of July, a committee was formed to consider and report on the recommendations contained in the report of the Lords' Committee on Metropolitan Medical Relief. That Committee having concluded its labours, invitations for a second meeting of the conference have been issued for January the 24th. The meeting will be held at the London School Board offices.

LORD WATERFORD, Chairman of the London Executive Committee of the Irish Distressed Ladies' Fund, make a special appeal for the Fund. He says: "I hope you will allow me again to appeal to the public through your columns. The charity has had large calls upon it during the past year, and I fear that there is nothing in the present condition of Ireland which is likely to mitigate the great distress among those ladies who have been so seriously affected by the agrarian troubles in that country. The charity is still in need of funds in order to continue its good work, and relieve the pressing and terrible distress that exists among a large number of ladies who, not many years ago, had every comfort at their command, but who are now almost entirely dependent for existence upon what they receive from this association. Last year, owing to the want of funds, it became necessary to reduce the pensions and allowances granted to aged and invalid ladies. During 1892 110 ladies who, from age or infirmity, are dependent upon this charity, being unable to work, have received monthly allowances according to the necessities of each case; 28 children are being educated; 119 invalids have received material help, and a large number of ladies have been employed in executing work which has been sold at the English and Irish depôts of the charity. Every case is thoroughly investigated, and great care taken to give assistance only to those who are really deserving. The Secretary will thankfully receive and acknowledge any subscriptions at the office of the Charity, 17, North Audley Street, London, W., which is also the depôt for the sale of the Irish distressed ladies' work."

WE have so often to condole with the hospitals on account of their financial difficulties that it is delightful to have so good a cause for congratulation. Baron de Hirsch evidently understands the luxury of giving to the full, and it would be difficult to find a parallel expenditure of the winnings of the Turf. Baron de Hirsch was the winner of £28,000 last year, and now the hospitals are reaping the benefit of his good fortune. Gifts have been made of £1,200 to the Children's Hospital, Great Ormond Street, of £700 to to the Royal Free, £300 to the Paddington Green Children's Hospital, £700 to the Great Northern Central, £200 to the Royal Sea Bathing Infirmary for Scrofula, £700 to the North-Eastern Hospital for Children, £700 to the Royal Hospital for Children and Women, and £700 to the Metropolitan Hospital.

A MEETING of the National Society for the Employment of Epileptics is to be held at the Mansion House on Wednesday, the 25th inst., at three p.m., under the presidency of the Lord Mayor. There are about 39,000 sane epileptics in Great Britain whose case is especially to be pitied, as they are able and willing to accept employment, which in the ordinary course it is not possible for them to obtain. There are but two homes for epileptics, that at Maghull, near Liverpool, and the Meath Home of Comfort at Godalming, the latter being for females only. The inadequacy of such provision is apparent. The Society propose establishing a colony for sane epileptics, and desire to purchase a piece of ground within easy reach of the metropolis for the purpose. The German epileptic colony has proved that such a scheme can be successfully carried out. We cannot doubt that the National Society will meet with hearty co-operation in their excellent endeavours, and that they will be so liberally supplied with means, that the establishment of the scheme on a much larger, and therefore much more effectual basis, than they modestly anticipate, will be possible.

A CORRESPONDENT to the *Chronicle* urges very strongly that more care should be exercised in the diagnosis of patients removed to fever hospitals. He quotes Dr. Cooney to the effect that as many as 462 mistakes were made during one year, attended with a large proportion of fatalities. Such statements are liable to disquiet the public in an unnecessary degree. This correspondent points out the public have the law in their own hands until the doctor has obtained a magistrate's certificate, should they desire to oppose removal. This fact generally known and acted upon whilst mistakes are obviated, more fatalities than those caused by occasional wrong diagnosis will probably occur. The Public Health Acts in their provisions for epidemic diseases have so markedly reduced the mortality of London that no impediments should be placed in the way of those who have to carry out the regulations. Consideration and tenderness towards the poor are qualities which we truly believe are steadily becoming more common amongst all classes of officials, and even now just complaints of its absence are few and far between.

